# DrDimont: explainable drug response prediction from differential analysis of multi-omics networks

**DOI:** 10.1093/bioinformatics/btac477

**Published:** 2022-09-18

**Authors:** Pauline Hiort, Julian Hugo, Justus Zeinert, Nataniel Müller, Spoorthi Kashyap, Jagath C Rajapakse, Francisco Azuaje, Bernhard Y Renard, Katharina Baum

**Affiliations:** Hasso Plattner Institute, Digital Engineering Faculty, University of Potsdam, Potsdam 14482, Germany; Hasso Plattner Institute, Digital Engineering Faculty, University of Potsdam, Potsdam 14482, Germany; Hasso Plattner Institute, Digital Engineering Faculty, University of Potsdam, Potsdam 14482, Germany; Hasso Plattner Institute, Digital Engineering Faculty, University of Potsdam, Potsdam 14482, Germany; Hasso Plattner Institute, Digital Engineering Faculty, University of Potsdam, Potsdam 14482, Germany; School of Computer Science and Engineering, Nanyang Technological University, Singapore 639798, Singapore; Genomics England, London EC1M 6BQ, UK; Hasso Plattner Institute, Digital Engineering Faculty, University of Potsdam, Potsdam 14482, Germany; Hasso Plattner Institute, Digital Engineering Faculty, University of Potsdam, Potsdam 14482, Germany

## Abstract

**Motivation:**

While it has been well established that drugs affect and help patients differently, personalized drug response predictions remain challenging. Solutions based on single omics measurements have been proposed, and networks provide means to incorporate molecular interactions into reasoning. However, how to integrate the wealth of information contained in multiple omics layers still poses a complex problem.

**Results:**

We present DrDimont, **D**rug **r**esponse prediction from **Di**fferential analysis of **m**ulti-**o**mics **n**e**t**works. It allows for comparative conclusions between two conditions and translates them into differential drug response predictions. DrDimont focuses on molecular interactions. It establishes condition-specific networks from correlation within an omics layer that are then reduced and combined into heterogeneous, multi-omics molecular networks. A novel semi-local, path-based integration step ensures integrative conclusions. Differential predictions are derived from comparing the condition-specific integrated networks. DrDimont’s predictions are explainable, i.e. molecular differences that are the source of high differential drug scores can be retrieved. We predict differential drug response in breast cancer using transcriptomics, proteomics, phosphosite and metabolomics measurements and contrast estrogen receptor positive and receptor negative patients. DrDimont performs better than drug prediction based on differential protein expression or PageRank when evaluating it on ground truth data from cancer cell lines. We find proteomic and phosphosite layers to carry most information for distinguishing drug response.

**Availability and implementation:**

DrDimont is available on CRAN: https://cran.r-project.org/package=DrDimont.

**Supplementary information:**

Supplementary data are available at *Bioinformatics* online.

## 1 Introduction

Personalized prediction of suitable medication is still a key task for computational approaches in clinical research. Meta-studies have shown that many drugs only work effectively in a fraction of patients (Leucht *et al.*, 2015), and consequences of failing treatment and adverse drug events can be severe. In recent years, more and more multi-omics profiles have become available that characterize disease phenotypes on a molecular level, especially in cancer, for example via the TCGA consortium (Chang *et al.*, 2013). Multiple layers of molecular data provide different perspectives and a higher resolution. Thus, a more fine-grained distinction between subgroups of patients is possible. At the same time, these data present the challenge of how to integrate them in order to derive meaningful predictions.

Different methods of omics integration have been proposed, distinguished frequently by when the omics layers are combined as well as by the goal of the analysis—molecular mechanism, patient clustering or other predictions such as drug response (Bersanelli *et al.*, 2016; Huang *et al.*, 2017; Picard *et al.*, 2021). Thereby, a genuine joint integration of the different layers has been considered advantageous (Cantini *et al.*, 2021; Picard *et al.*, 2021). Joint dimensionality reduction, e.g. via ICA or MOFA (Argelaguet *et al.*, 2018; Sompairac *et al.*, 2019), has been proposed to find relevant features from combined multi-omics data. They have been benchmarked for use with cancer data (Cantini *et al.*, 2021). However, the reduced meta-genes are difficult to interpret and hinder direct conclusions on drug action.

The fact that molecules do not act separately, but in their network context led to an alternative joint integration strategy: network-based approaches enable consideration of interactions between entities and have been specifically applied to multi-omics data (Demirel *et al.*, 2021; Lee *et al.*, 2019; Recanatini and Cabrelle, 2020; Yugi *et al.*, 2016). Networks other than purely molecular heterogeneous networks have been proposed, containing, e.g. diseases, drugs or cell lines as nodes (Stanfield *et al.*, 2017; Zhang *et al.*, 2018a), and patient similarity networks derived from molecular data (Wang *et al.*, 2014). A plethora of methods have been suggested to establish and use molecular networks to find relevant disease genes (Dimitrakopoulos *et al.*, 2018; Ogris *et al.*, 2021; Peng *et al.*, 2017; Schulte-Sasse *et al.*, 2021). Moreover, interactions between molecules are one of the key readouts of drug action: drugs interfere most frequently with the function of the targets they bind to, hampering their ability to interact with other molecular players instead of affecting their overall abundance (Pinto *et al.*, 2014). Therefore, considering interactions in molecular networks (Bartel *et al.*, 2015; Koh *et al.*, 2019; Sambaturu *et al.*, 2020) is highly promising for drug response prediction.

Systematic drug response measurements are available, especially for cancer cell lines (Barretina *et al.*, 2012; Rees *et al.*, 2016; Yang *et al.*, 2013), and therefore, drug response prediction has been performed for this *in vitro* setting (Ding *et al.*, 2018; Park *et al.*, 2022; Zhang *et al.*, 2018a). The question remains how these results can be transferred to clinically relevant predictions for patients. Some transfer learning approaches have been proposed (Geeleher *et al.*, 2017; Webber *et al.*, 2018), but these still rely on prior systematic measurements of drug response in conditions comparable to the condition of interest. Methods of artificial intelligence are being advanced (Azuaje, 2019), but the explanation of their results is frequently challenging. A viable strategy is to assess differential response to a baseline patient group phenotype. This technique has been proposed to query differential co-expression (Bhuva *et al.*, 2019; Matsui *et al.*, 2021), gene ranking (Richard *et al.*, 2020) or networks or paths (Ideker and Krogan, 2012; Sambaturu *et al.*, 2020), but not differential drug response yet.

Here, we present our new approach for **D**rug **r**esponse prediction from **Di**fferential analysis of **m**ulti-**o**mics **n**e**t**works, DrDimont, which unites the following key points: (i) multi-omics data are jointly integrated, including data such as metabolomics, (ii) condition-specific molecular networks are built, (iii) prior and domain-specific knowledge on molecular interactions can be leveraged, (iv) the focus is on interactions between molecules as the most common mode of drug action, (v) differential analysis between conditions enables unsupervised predictions in clinically relevant settings and (vi) the predictions are explainable as their underlying molecular characteristics can be retrieved.

We describe DrDimont and apply it to a breast cancer dataset combining transcriptomics, proteomics, phosphosite and metabolomics measurements. We compare DrDimont’s differential drug response predictions to ground truth from cell line measurements, and to alternative approaches. We investigate the impact of different measurement layers on the prediction quality. Finally, we showcase an example of how DrDimont explains results down to the molecular level.

## 2 Materials and methods

### 2.1 Differential predictions with DrDimont

DrDimont provides a framework to leverage condition-specific, weighted heterogeneous networks for differential analysis between two conditions. It builds purely molecular networks with nodes encoding entities within a cell, such as proteins, mRNAs, metabolites and their interactions from both multi-omics data and prior information on interactions from databases.

**Fig. 1.**
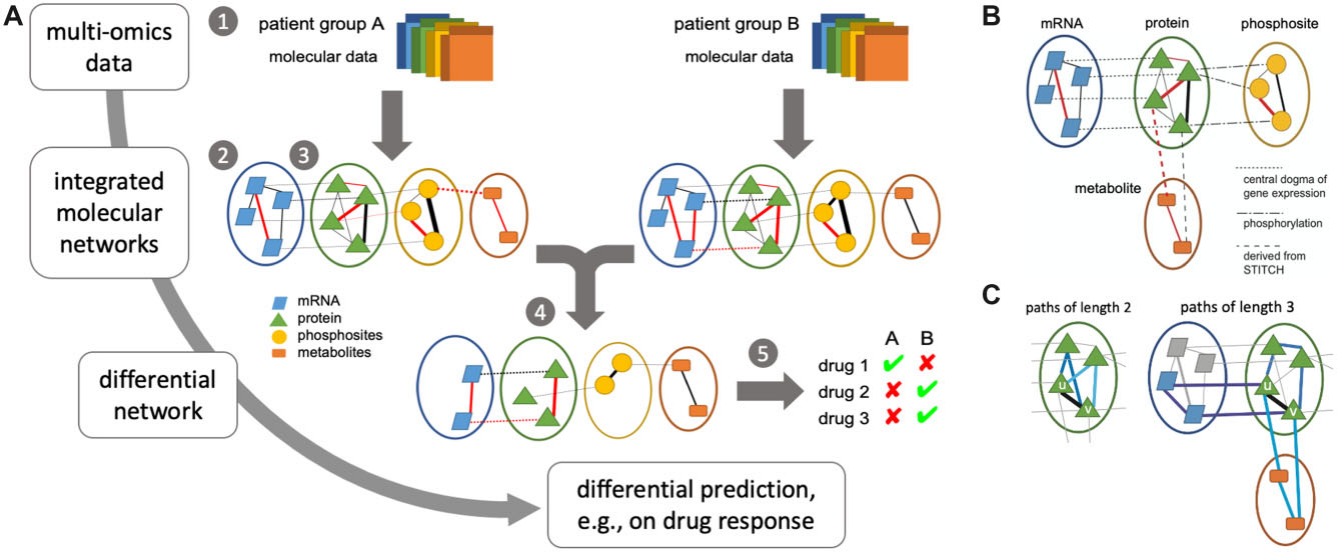
DrDimont’s pipeline for integrated, network-based analysis. (**A**) Multiple molecular data layers for two conditions are compared, e.g. cancer data (1). They are used to derive condition-specific correlation-based single-layer networks (2) that are combined into integrated molecular networks using prior information (3). The differential network is derived from the condition-specific networks (4) and captures altered interaction strengths. Differences, e.g. in drug response, are predicted from the differential network (5). (**B**) Example for the generation of an integrated, multi-layer weighted network with the protein network as central layer. Within-layer interactions are correlation-based from measurements. Different layers are connected by prior information, its type is encoded by the dashed lines. Edges can have negative as well as positive edge weights (indicated by different line colors), thicker lines indicate larger edge weights. (**C**) Integrated interaction scores. Edge weights are replaced by the average over the strengths of alternative paths, thus generating a local enhancement exploiting network structure (see Equation (1)). For example, for the edge connecting nodes *u* and *v*, a selection of alternative paths is marked by thicker edges: paths of length two (left), or paths of length three (right)

An overview of the pipeline provided in the DrDimont framework is shown in Figure 1. DrDimont requires quantified molecular data such as RNAseq or protein data of several samples as input. For differential analysis, data for two different groups of samples or patients (‘conditions’) are needed. Each molecular data input layer is transformed into condition-specific weighted, single-layer networks by correlation of the molecular entities. Then, based on a user-defined structural requirement (see Section 2.1.1), the networks are reduced keeping edges with high weights only. As shown for an example in Figure 1B, the single-layer networks are combined into multi-layer networks based on user-defined inter-layer connections. Thereby, prior knowledge on interactions from databases can be incorporated. The two condition-specific multi-layer networks are further integrated by computing integrated interaction scores, as shown in Figure 1C. These propagate local neighborhood information to the edge weights and thus avoid too strong of an impact of single edges. From the two integrated networks a differential network is computed by contrasting the edge weights of the condition-specific networks. The differential network is employed to calculate differential drug response scores based on the differential edges in vicinity to a drug’s targets. We will describe details for each step in the following.

#### 2.1.1 Single-layer network generation

For each data layer and each group, complete weighted networks are generated where each node represents one type of molecule, e.g. one specific mRNA or protein. The weight of an edge between any two nodes of one layer is derived from the correlation between the abundance measurements for the nodes over all samples in the group, e.g. using Spearman’s or Pearson’s correlation. If not stated otherwise, we employed Spearman’s correlation in order to avoid strong impact of outliers and to account for non-linearity in abundance relationships. In case of missing values, pairwise complete observations were used for correlation.

The correlation-based networks are reduced and only the edges with the largest absolute weights are kept. Reduction thresholds are determined (i) by a desired average degree of the network nodes, (ii) by a desired average network density or (iii) by maximizing the scale-freeness of the network (WGCNA, pickHardthreshold function, Langfelder and Horvath, 2008). If not stated otherwise, we used this latter topological criterion here and adapted the goodness of fit to a scale-free network, *R*^2^, to have similar-sized networks for the compared groups. See the Supplementary Material for the impact of *Alternative reduction methods*.

#### 2.1.2 Heterogeneous multi-layer network construction

DrDimont connects single-layer networks for each group separately based on the node names (see Fig. 1B). First, nodes from different layers with identical names can be connected with edges of the same user-defined weight (e.g. of value one). This allows exploiting the dogma of gene expression, i.e. connecting an mRNA to its corresponding protein, or a protein to its corresponding phosphosites. Representing further relationships is possible such as methylated promoter regions on the DNA to the corresponding target genes or mRNAs. Second, pairs of node names from two different layers can be entered by the user. These nodes will then be connected. The edge weights can be again fixed or derived from prior information, for example, to connect the protein and the metabolite layer using data from a database such as STITCH (Szklarczyk *et al.*, 2016) (see Supplementary Material, *Metabolite-protein interactions from STITCH* for details).

#### 2.1.3 Integrated interaction scores

DrDimont uses a novel, semi-local integration scheme to reduce the impact of single edge weights in the condition-specific networks. Thereby, the weights of alternative paths between nodes are taken into account (see Fig. 1C). Edge weights in the heterogeneous multi-layer network can be replaced by their integrated interaction scores, that is, the average strength of these alternative paths. For an edge connecting nodes *u*, *v*, we define this score, $s_{u,v}$, as the sum of average strengths of alternative paths connecting *u* and *v* over the considered path lengths, i.e.

(1) $$s_{u,v}=\sum_{l=1}^{L}\frac{1}{\left|{\text{path}}_{l|u,v}\right|}\sum_{k\in {\text{path}}_{l|u,v}}\prod_{e\in k}\text{weight(e)}.$$

Thereby, ${\text{path}}_{l|u,v}$ is the set of paths of length *l* between nodes u and v, L is the maximal length of considered paths, and weight(e) is the edge weight of an edge e. For a path k of length l that is connecting nodes $x_{0},\ldots ,x_{l}$ in this order, k is determined by its set of contributing edges $k=\{(x_{0},x_{1}),(x_{1},x_{2}),\ldots (x_{l-1},x_{l})\}$, and all their edge weights are multiplied to determine the path strength of k. For edge weights ranging from –1 to 1 as in usual correlation measures, integrated interaction scores can range from −L to L. If not stated otherwise, we used L* *=* *3. In order to reduce DrDimont’s run time for large networks, the integrated interaction scores are only computed for edges incident to user-provided drug targets by default (see also Section 2.1.5).

#### 2.1.4 Differential network

DrDimont generates the differential network by computing the difference between integrated interaction scores of all edges of the group-specific heterogeneous multi-layer networks. Edges that only appear in one of the group’s networks are considered to have a weight of zero in the other networks and will be part of the differential network. Nodes that are part of any of the two networks are thus included in the differential network.

#### 2.1.5 Differential drug response score

The differential network is used for DrDimont’s differential drug response prediction. To derive a prediction for a drug, drug targets have to be known. The drug targets are identified within the differential network. The absolute value of the mean (default, or the median) of the weights of all edges incident to the drug targets is used as a drug’s differential drug response score. The differential drug response score is the main output of DrDimont and provides a prioritization (ranking) of the drugs. If not stated otherwise, we used proteins as drug targets. However, in principle, nodes from any input molecular layer can be defined as drug targets.

DrDimont’s implementation details and its settings for heterogeneous network construction are provided in the Supplementary Material.

### 2.2 Molecular breast cancer dataset preparation

We used a breast cancer dataset from patient tumors with mRNA (measured via RNAseq) from TCGA, proteomics and phosphosites (measured via mass spectrometry) from CPTAC (Mertins *et al.*, 2016). We combined these data with metabolite data from two other studies (Budczies *et al.*, 2013; Terunuma *et al.*, 2014). The mRNA data and clinical annotations were downloaded from TCGA via RTCGA (Kosinski and Biecek, 2016). We obtained the estrogen receptor status, negative (ER−) or positive (ER+), for each sample from the clinical annotations (for sample counts, see Supplementary Table S1).

We disregarded mRNAs with more than 90% of zero measurements over the samples within a condition. Proteins and phosphosites with more than 20% of missing values over the samples of a condition were removed. There were no missing values for the metabolite data as imputation had been done by the respective authors prior to publication of the data. If not stated otherwise, the genetic features (mRNA, protein, phosphosites) were reduced to a subset of 5579 known cancer-related genes (Repana *et al.*, 2019) and drug targets from DrugBank (Wishart *et al.*, 2017).

### 2.3 Drug targets, drug response ground truth, performance

We retrieved data for 40 breast cancer cell lines (26 ER−, 14 ER+) from the Cancer Therapeutics Response Portal (CTRP) (Rees *et al.*, 2016), in particular drug sensitivity, compound data and drug target information for 481 drugs. We used estrogen receptor status annotation from the DepMap portal (DepMap, Broad, 2021). We employed data for a drug if it was measured at least three times for each condition. We determined the differential drug response between ER+ and ER− for each drug by Mann–Whitney *U* tests comparing sensitivity in ER+ cell lines vs. sensitivity in ER− cell lines obtaining ground truth for 477 drugs with the *P*-value as ranking (see Supplementary Fig. S1 for the effect size instead of *P*-value). For performance assessment, we report Spearman’s correlation between predicted and ground truth drug ranking, and the *P*-value of the correlation. Only drugs for that the analysis in question delivered a prediction were used; their numbers are indicated accordingly. In particular, drugs lacking known drug targets were disregarded.

Receiver operating characteristic (ROC) curves were generated by comparing prediction-derived to ground-truth derived binary drug classifications (see Supplementary Material for details). We indicate which fixed ground-truth threshold was employed. We report the area under the ROC curve (AUC). For partial AUC (pAUC), we compute the AUC for false positive rates between 0 and 0.1. High pAUC values signify enriched true predictions among the top ranked drugs.

Data preparation, visualization and result analysis were performed using R, version 4.1.0 (R Core Team, 2021).

### 2.4 Alternative prediction methods

To assess DrDimont’s performance, we implemented two alternative differential drug response prediction approaches.

#### 2.4.1 PageRank of drug targets in the differential network

We used igraph (Csardi and Nepusz, 2006) to compute the weighted PageRank (Brin and Page, 1998) of all potential drug targets in the undirected differential network from DrDimont. Therein, we employed the absolute differential edge weights (and not differential integrated interactions scores). The mean weighted PageRank over all drug targets generated the differential drug response ranking.

#### 2.4.2 Differential protein expression

We employed the Differential Enrichment analysis of Proteomics data (DEP) pipeline (Zhang *et al.*, 2018b) to assess differential protein expression between ER+ and ER− conditions. Therein, after a variance-stabilizing transformation, *limma* is applied. This single-layer approach delivered a ranking of the drugs with respect to differential drug prediction from differential protein expression of all drug targets of a drug using the minimal multiple-testing adjusted *P*-value.

## 3 Results

We will now showcase results of DrDimont for a multi-omics dataset and assess its performance with measured ground truth. Then, we will compare it to two alternative differential drug response prediction approaches. Furthermore, we will describe the impact of including different data layers in DrDimont’s analysis, and end with an illustration of the level of explainability that DrDimont provides.

### 3.1 Evaluation of DrDimont on breast cancer stratified by estrogen receptor status

We investigated molecular data of breast cancer patients stratified by estrogen receptor status. The ER status is highly prognostic, with ER+ patients having a better prognosis than ER− patients.

**Fig. 2.**
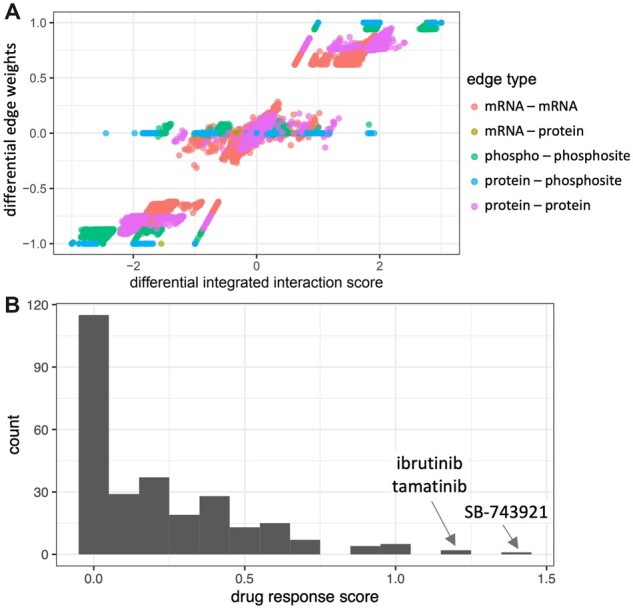
DrDimont’s integrated interaction scores and differential drug response predictions for the breast cancer dataset. (**A**) Differential edge weights are compared to the differential integrated interaction scores of DrDimont for each of the five edge types (colored). We contrasted ER− versus ER+, i.e. negative scores correspond to stronger inhibitory interactions or less strong positive interactions in ER− compared to ER+. Integrated interaction scores enable distinguishing edges that have a zero differential edge weight, but overall, they show a high correlation (Spearman’s *ρ* 0.967). (**B**) Histogram of DrDimont’s differential drug response scores of 275 drugs for which drug targets occur in our multi-layer networks. Half of the drugs are predicted with a differential response of varying value

We first considered a multi-omics dataset that provides ER-stratified patient data containing transcriptomics measurements via RNAseq (from TCGA), and proteomics and phosphosite data from mass spectrometry-based measurements (from CPTAC, Mertins *et al.*, 2016). We also included metabolomics data from other studies later (Budczies *et al.*, 2013; Terunuma *et al.*, 2014). We show the properties of the differential integrated network generated with DrDimont for this dataset (without metabolomics) in Figure 2A. The differential integrated interaction scores are correlated with the differential edge weights, but the former allow for broader distributions. They are taking alternative paths into account and thereby propagate the information from the local neighborhood to the respective edges. In particular, the edges derived from prior information (mRNA–protein, protein–phosphosite) benefit from this procedure: Their edge weights are not condition-specific and therefore, their differential edge weights cluster around minus one, zero, and one. In contrast, their differential integrated interaction scores are spread out and differences between conditions are resolved in greater detail.

For our dataset, DrDimont provides drug response scores for 275 drugs with drug targets from CTRP (Rees *et al.*, 2016) (see Fig. 2B), the majority were differentially predicted to some degree. Only seven drugs have a drug response score of one or higher. Top differentially predicted drugs were SB-743921, that is known to have a stronger effect in ER− cell lines (Zhu *et al.*, 2016), ibrutinib and tamatinib.

**Fig. 3.**
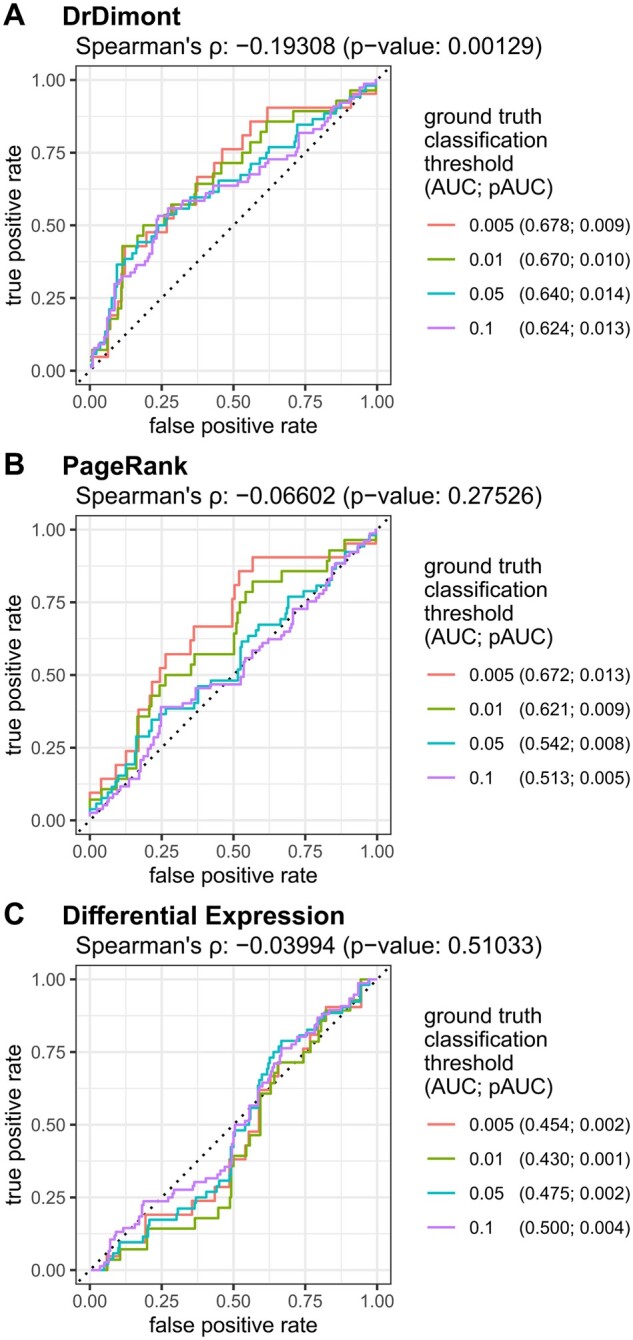
ROC curves for DrDimont’s and alternative methods’ prediction performance on the breast cancer dataset. (**A**) DrDimont’s differential drug response. Different ground truth thresholds were used where drugs below the threshold were considered as true positives and the others as true negatives. For 275 drugs, DrDimont provided differential drug response scores that were used for drug ranking and comparison to ground truth classification. (**B**) Weighted PageRank of drug targets for differential drug response prediction, for 275 drugs, computed on DrDimont’s differential network. (**C**) Differential expression of drug target proteins for differential drug response prediction. The differential expression yielded predictions for 274 drugs. In the legends, values in brackets denote AUCs, pAUCs at given ground truth thresholds. The respective Spearman’s *ρ* and the *P*-value are given at the top of each figure. Black dotted lines indicate theoretical ROC curves for random predictions

We compared the results of DrDimont for our dataset to the ground truth of the drugs from CTRP in a ROC performance analysis (see Fig. 3A). DrDimont’s drug response scores were used as drug ranking for computation of true and false positive rates of the prediction. The AUC for a ground truth threshold of 0.01 was 0.67 (see legend of Fig. 3A) which is considerably higher than for random prediction (AUC 0.5). DrDimont’s multi-omics data-based drug response scores showed a significant correlation to the CTRP-based ground truth (Spearman’s *ρ* −0.19, *P*-value 0.001). The correlation is negative since the ground truth is based on *P*-values, i.e. lower values correspond to a likelier differential response. What is more, the pAUC that takes only highly ranked drugs into account gives values decisively higher than expected from a random prediction (up to 0.014 compared to 0.005). Thus, we find DrDimont to be predictive of differential drug response, and it especially enriches positive hits among the top ranked results.

### 3.2 Comparison to alternative differential drug prediction approaches

DrDimont’s approach focuses on differential interactions of drug targets in the molecular network for differential drug response predictions. However, frequently, rather the node properties are considered for predictions. Therefore, we compare DrDimont’s performance to two alternative approaches for deriving differential drug response: (i) using the weighted PageRank algorithm in DrDimont’s differential network to score drug targets, and (ii) differential expression of drug targets. PageRank detects nodes with high importance in a network (hubs) and could thus identify drug targets that are particularly altered between conditions from the differential network. Differential expression has been considered relevant for drug action and has been used especially for predictions of drug responsiveness of different tissues. We based our estimations on the differential protein expression of a drug’s targets because most frequently these are the drug’s binding partners rather than, e.g. mRNA molecules.

For our breast cancer dataset, the weighted PageRank for drug targets yielded predictions better than random, especially for stringent ground-truth thresholds (AUCs > 0.5, Fig. 3B). Spearman’s correlation with the ground-truth ranking was less pronounced than for predictions with DrDimont and not significant (−0.06, *P*-value 0.27), and pAUCs were below random (pAUC < 0.005). The predictions based on differential protein expression were close to random classification (AUC ≤ 0.5). Also the correlation between prediction and ground truth ranking was small and insignificant (Spearman’s *ρ* −0.03, *P*-value 0.5, see Fig. 3C). Thus, both the PageRank-based and the differential expression-based drug response predictions performed worse than DrDimont on the breast cancer dataset.

### 3.3 Influence of different molecular layers

We analyzed which molecular layers are most relevant for DrDimont’s drug response prediction performance. Therefore, we considered mRNA, protein, phosphosite data as before, as well as the two metabolomics datasets (see Table 1, and Supplementary Fig. S4 for the ROC curves). DrDimont performed best according to AUC and Spearman’s correlation when employing only the proteomic layer in the analysis. However, DrDimont could only provide predictions for 116 drugs in this setting; the remaining drugs lacked edges for all of their drug targets in the network thereby resulting in no drug response score (see Supplementary Fig. S5 for a further characterization). Compared to the default setting with all three data layers (no metabolomics), using protein and phosphosite data together resulted in only slight changes in AUC and correlation, but more than doubled the pAUC from 0.01 to 0.024 (best value). DrDimont performed worst in terms of AUC and correlation when using only the mRNA and the protein layers together. Applying DrDimont using only the phosphosite layer resulted in a relatively high pAUC of 0.017. Surprisingly, adding the two different metabolite datasets to the analysis with DrDimont showed consistently worse results than without metabolites, but affected the results for the setting containing all other three data layers least. We conclude that the proteomic and phosphosite data layers contribute most to DrDimont’s differential drug response prediction for this dataset.

**Table 1.** Influence of different molecular data layers on DrDimont’s drug response prediction performance

Included layers	#drugs	AUC	pAUC	corr.	*P*
mRNA, proteins, phosphosites	275	0.670	0.010	−0.193	**0.001**
+ metabolites B	275	0.634	0.009	−0.179	0.003
+ metabolites T	275	0.634	0.007	−0.172	0.004
mRNA, proteins	275	0.552	0.008	−0.058	0.338
mRNA	216	0.574	0.001	−0.114	0.093
proteins, phosphosites	190	0.668	**0.024**	−0.188	0.009
+ metabolites B	213	0.626	0.008	−0.156	0.023
+ metabolites T	194	0.643	0.007	−0.145	0.034
proteins	116	**0.677**	0.021	**−0.253**	0.006
+ metabolites B	178	0.479	0.008	−0.035	0.639
+ metabolites T	136	0.509	0.008	−0.058	0.439
phosphosites	115	0.585	0.017	−0.179	0.055

*Note*: T: Terunuma metabolomics, B: Budczies metabolomics. The respective number of drugs with differential drug response predictions can change between approaches because the established molecular networks differ and drugs without any edges incident to their drug targets do not receive a differential drug response score. We indicate the AUC and pAUC for a ground truth threshold of 0.01, and Spearman’s correlation (corr.) and its *P*-value (*P*).

### 3.4 Explainable results

**Fig. 4.**
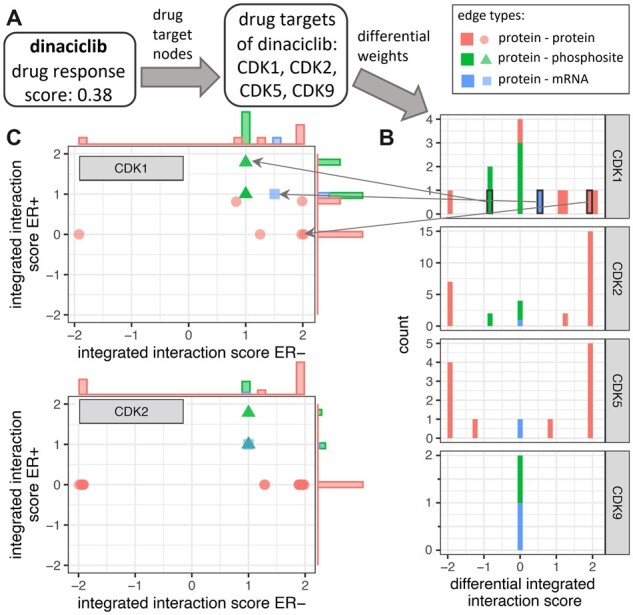
DrDimont delivers explainable predictions. Given a drug (example: dinaciclib), the differential drug response score derived from DrDimont can be traced back to the input layers. (**A**) Dinaciclib’s drug targets (CDK1, CDK2, CDK5, CDK9) are identified in the differential network. (**B**) The distribution of differential integrated interaction scores of the drug targets’ incident edges can be retrieved (stacked histogram). Many edges that are differential occur for CDK2 and CDK5. (**C**) The differential integrated interaction score of an edge can be related to the interaction strength in each condition (here: ER+ versus ER−, for CDK1 and CDK2). Bars to the sides resolve edge counts. Boxes and arrows mark respective values in (**B**) and (**C**) for three selected edges incident to CDK1. Different edge types are marked by colors

An asset of DrDimont is that predictions can be directly associated to molecular differences between subgroups. Given a specific drug response score for a certain drug, it can be traced back which drug target is especially different between the compared conditions, as well as which connections of the drug targets are the cause of the observed differences. We show this in an example for the drug dinaciclib, see Figure 4 (see Supplementary Fig. S7 for additional analyses). Dinaciclib’s four reported drug targets CDK1, CDK2, CDK5, CDK9 and their incident edges can be identified both in the differential network as well as in the network for each condition. We find that CDK2 (and CDK5, not shown) has stronger interactions with other proteins in ER− than in ER+, whereas the interactions between proteins and their phosphosites are equally strong or stronger for CDK1 and CDK2 in the ER+ group. CDK9 shows no interaction differences between conditions. Specific differently interacting proteins and phosphosites can be also retrieved. This allows a deeper investigation by domain experts.

## 4 Discussion

We introduce DrDimont as a flexible framework for network-based differential analysis and drug response prediction. It builds condition-specific molecular networks from multi- or single-omics, and no matched samples are required. In addition, DrDimont outperforms differential expression-based and PageRank-based approaches for drug response prediction. It provides an explainable framework to trace contributions of single molecular alterations.

We find the protein and phosphosite layers to be most informative for drug prediction, especially for identifying top ranked drugs. Biologically, this seems reasonable because drugs mainly act on proteins where they interfere with their cellular functions (Pinto *et al.*, 2014). Further, these functions are frequently modulated by post-translational modifications such as phosphorylations (Dittmar *et al.*, 2019). Despite insights on the relevance of metabolomics in combination with other omics data for disease and in particular for cancer (Ortmayr *et al.*, 2019; Pinu *et al.*, 2019), we do not find evidence supporting that in our analyses. A possible reason could be that while all other omics data are from the same study and experimental conditions, the metabolomics measurements stem from different studies possibly adding extra noise to the integrated results. In addition, reliable measurement of metabolite abundances is more difficult in a patient treatment setting because metabolites are degraded extremely rapidly also within extracted tumor biopsies. Further improvement may be achieved by reducing the number of the other layers’ features (nodes) for better balance of their sizes. Additionally, other intra- and inter-layer connection approaches, such as derived from protein–protein interaction or metabolic networks, might be viable options to explore within the DrDimont framework.

DrDimont’s combined molecular networks can be retrieved and employed for the user’s own analysis approaches, for example using network embedding (Pio-Lopez *et al.*, 2021), exploration by random walks (Valdeolivas *et al.*, 2019) or diffusion-based methods (Di Nanni *et al.*, 2020). A strength of DrDimont is that its networks can also be tracked to provide molecular explanations for the predictions and thereby enable targeted biological follow-ups for in-depth investigation. In addition, DrDimont allows the inclusion of molecules with unknown function and low abundances. This makes the approach particularly interesting also for less well characterized organisms such as fungi.

Applying DrDimont to compare more refined subgroups would be interesting, for example, resolving effects of other hormone receptors in breast cancer such as progesterone or HER2. Other groups to compare could be pre- and post-chemotherapeutic patients (Park *et al.*, 2020), or applying general sample classification before analysis with DrDimont.

A limitation of our network-based drug response prediction is that it relies on the quality of known drug targets. Some drugs are less well characterized than others. Taking this uncertainty into account and increasing the resolution on the mode of action of drugs for the input data (Parvizi *et al.*, 2020) could be opportunities for improving DrDimont’s prediction results. Moreover, real ground truth for our case is difficult to obtain: applying different drugs to patients and monitoring their outcome cannot be performed, of course, due to ethical reasons and standard of care. Here, we use cell line measurements as a surrogate that differ in quality, taking trade-offs into account. Overall, differential drug response prediction is a difficult problem that also results in contradicting ground truth measurements (see Supplementary Fig. S6), and thus, comparably low AUCs are not surprising. The pAUC that measures enrichment of correct predictions in top-differential drugs achieves high values. Other directions such as relying on extended patient-derived xenograft-based drug response studies and analyses (Gao *et al.*, 2015) could be taken.

DrDimont is a flexible tool for subgroup-specific and comparative predictions with an explainable framework, and we envision that it contributes with its proof-of-principle to improving the clinical decision process in the future.

## Supplementary Material

btac477_Supplementary_Data

## Data Availability

The data underlying results of this article were accessed from Proteomic Data Commons, https://pdc.cancer.gov/pdc/, PDC000173 (file TCGA_Breast_BI_Proteome.itraq.tsv) and PDC000174 (file TCGA_Breast_BI_Phosphoproteome.phosphosite.itraq.tsv); from TCGA, http://gdac.broadinstitute.org/runs/stddata__2016_01_28/data/BRCA/20160128/ (file BRCA.clin.merged.txt within gdac.broadinstitute.org_BRCA.Merge_Clinical.Level_1.2016012800.0.0.tar.gz, file BRCA.uncv2.mRNAseq_RSEM_normalized_log2.txt within gdac.broadinstitute.org_BRCA.mRNAseq_Preprocess.Level_3.2016012800.0.0.tar.gz); from DrugBank, https://go.drugbank.com/releases/5-1-8#protein-identifiers (file all.csv); from the Network of Cancer Genes, http://ncg.kcl.ac.uk/download.php (NGCv7.0); from STITCH, http://stitch.embl.de/cgi/download.pl (files chemical.aliases.v5.0.tsv.gz, 9606.actions.v5.0.tsv.gz, 9606.protein_chemical.links.detailed.v5.0.tsv.gz); from the DepMap Portal, https://depmap.org/portal/ (file sample_info.csv from 21Q4) and from journal articles as indicated in the text.
