# Supplementary material for: DrDimont: explainable drug response prediction from differential analysis of multi-omics networks
Source: Bioinformatics. 2022 Sep 18;38(Suppl 2):ii113–9. doi: 10.1093/bioinformatics/btac477 (PMC9486584; doi:10.1093/bioinformatics/btac477)
Supplement: btac477_Supplementary_Data [file btac477_supplementary_data.pdf]

Supplementary Information - DrDimont: Explainable drug response prediction from differential analysis of multi-omics networks

Pauline Hiort Julian Hugo Justus Zeinert Nataniel Müller
Spoorthi Kashyap Jagath C. Rajapakse Francisco Azuaje Bernhard Y. Renard
Katharina Baum

2022

Contact: katharina.baum@hpi.de

Contents

1 Details on methods and settings **2**
1.1 DrDimont's implementation details . . . 2
1.2 DrDimont's settings for heterogeneous network construction . . . 2
1.3 Metabolite-protein interactions from STITCH . . . 2
1.4 Performance assessment via ROC, AUC, pAUC . . . 2
1.5 Sample counts, network sizes, reduction thresholds . . . 3

2 Additional analyses - Supplementary Figures **4**
2.1 Effect size for the ground truth . . . 4
2.2 Alternative reduction methods . . . 5
2.3 Integrated interaction score settings and differential drug prediction method . . . 6
2.4 ROC curves for data layer ablation study . . . 7
2.5 Drugs lacking predictions for data layers . . . 8
2.6 Alternative ground truth measurements from GDSC . . . 9
2.7 Explanations for DrDimont's top-predicted drugs and single drug targets . . . 10
2.8 Performance for unreduced and other datasets . . . 11

1 Details on methods and settings

For general data analysis and visualization, we used R, version 4.1.0 [19]. In addition, we employed the R packages ggplot2 [23], ggextra [2], dplyr [25], tidyr [26], and Hmisc [7].

1.1 DrDimont's implementation details

DrDimont is implemented as an R package and employs python [18]. The package was tested with R version 4.1.3 and python 3.9.6. R packages employed by DrDimont include igraph [5], WGCNA [12], dplyr [25], tibble [15], tidyr [26], stringr [24], Rfast [17], readr [27], magrittr [3], rlang [9], rmarkdown [1], knitr [28], utils, and stats. Python packages used are numpy (version 1.20.3) [8], tqdm [6], python-igraph (version 0.9.9) [5] and ray (version 1.6.0) [14]. DrDimont is available on CRAN: https://cran.r-project.org/package=DrDimont.

1.2 DrDimont's settings for heterogeneous network construction

For the analysis of our datasets, DrDimont combined the mRNA, protein and phosphosite layers based on the gene names using edge weights of 1, i.e., an mRNA was linked to its protein, and a protein was linked to its phosphosites. The metabolites were linked to the proteins employing STITCH [21] interactions with confidence scores as edge weights (see *Metabolite-protein interactions from STITCH*).

1.3 Metabolite-protein interactions from STITCH

Since combining the metabolite and protein or other genetic layers is not as straightforward, we downloaded all chemical-protein interactions from STITCH [21] for *Homo sapiens*. We restricted them to high-confidence interactions with a STITCH combined score > 900 and the sum of STITCH sub-scores for database and experimental evidence > 800. Interaction weights between proteins and metabolites were set as the combined STITCH score divided by 1000, and interactions were assigned a negative weight if they were identified as inhibition. We identified an interaction as inhibition if high-confidence evidence (score > 800) was given in the STITCH table of mode of actions. If both activation and inhibition were indicated for an interaction, we used the mode with a higher confidence. For the proteins, we mapped the STITCH ensembl_peptide_id to gene symbols using biomaRt. We used the aliases file from STITCH for identifying metabolites in our datasets based on Pubchem IDs, ChEBI IDs, KEGG IDs and metabolite names (in that order). The thus identified stereo chemical IDs for the aliases were used to match the chemical IDs of the STITCH interactions to the metabolite names given in the case study datasets.

1.4 Performance assessment via ROC, AUC, pAUC

For receiver operating characteristic (ROC) analysis, a threshold for the ground truth ranking was applied to define a binary classification of drugs that have a differential response (low p-value, true positives) or have no differential response (high p-value, true negatives) in the ER- vs. ER+ cell lines. For the ROC curves of a predicted drug ranking, thresholds were varied to obtain predicted binary drug classifications. These were compared with the ground truth binary classification to derive false positive and true positive rates. The area under the ROC curve (AUC) was computed by numerical integration. The partial AUC (pAUC) was computed as area under the ROC curve between false positive rates of 0 and 0.1. Thus, random predictions yield pAUCs of 0.005. High pAUC values signify enriched true predictions among the top ranked drugs.

1.5 Sample counts, network sizes, reduction thresholds

Table S1: Number of samples, nodes, reduced edges and DrDimont settings for network reduction per dataset, group and molecular layer. The network reduction parameters used in DrDimont were applied to both groups that we compared respectively. 'red. TCGA' denotes the TCGA + CPTAC dataset with reduced genetic features (our default) and 'TCGA' the unreduced dataset. The other two datasets, Krug [11] and cell lines [13, 16, 10], are described further in section *Performance for unreduced and other datasets.* The datasets Budczies [4] and Terunuma [22] are the metabolomics datasets.

	Group	red. TCGA	TCGA	Krug	Cell lines	Budczies	Terunuma
mRNA							
samples	ER-	237	237	34	38	-	-
samples	ER+	806	806	78	20	-	-
nodes	ER-	4120	18321	21015	18272	-	-
nodes	ER+	4120	18321	20747	17938	-	-
reduced edges	ER-	21630	262787	86283	61703	-	-
reduced edges	ER+	25036	138134	30498	34941	-	-
R^2	both	0.85	0.85	0.87	0.89	-	-
WGCNA threshold range	both	0.4-0.8	0.55-0.8	0.56-0.8	0.55-0.9	-	-
protein							
samples	ER-	36	36	34	23	-	-
samples	ER+	68	68	78	9	-	-
nodes	ER-	2917	8430	9018	7443	-	-
nodes	ER+	2920	8459	9011	7157	-	-
reduced edges	ER-	17634	81777	40621	83959	-	-
reduced edges	ER+	6073	151712	55639	74741	-	-
R^2	both	0.84	0.84	0.86	0.85	-	-
WGCNA threshold range	both	0.7-0.8	0.7-0.85	0.65-0.8	0.7-0.95	-	-
phosphosites							
samples	ER-	36	36	34	-	-	-
samples	ER+	67	67	78	-	-	-
nodes	ER-	4942	12290	18222	-	-	-
nodes	ER+	4942	12223	17885	-	-	-
reduced edges	ER-	15109	34377	57845	-	-	-
reduced edges	ER+	80674	10960	23468	-	-	-
R^2	both	0.95	0.94	0.97	-	-	-
WGCNA threshold range	both	0.7-0.95	0.85-0.95	0.7-0.9	-	-	-
metabolites							
samples	ER-	-	-	-	30	41	34
samples	ER+	-	-	-	18	143	33
nodes	ER-	-	-	-	225	162	342
nodes	ER+	-	-	-	225	162	307
reduced edges	ER-	-	-	-	1723	2192	8639
reduced edges	ER+	-	-	-	1322	2335	6031
R^2	both	-	-	-	0.6	0.7	0.7
WGCNA threshold range	both	-	-	-	0.6-0.8	0.2-0.95	0.2-0.95

2 Additional analyses - Supplementary Figures

2.1 Effect size for the ground truth

In the manuscript, we derived the ground truth by contrasting breast drug response measurements from multiple ER-stratified cancer cell lines from CTRP [20] with a Mann-Whitney-U test. We used the p-value as ground truth readout: the smaller the p-value, the more differential the drug response between conditions. Here, we investigated the impact of using the effect size instead of the p-value. The effect size is defined as $|z|/\sqrt{N}$, with N the sample size, i.e. the number of measured breast cancer cell lines for a drug, and z the z-score of the test statistic. We performed ROC analysis for DrDimont's predictions, prediction by PageRank, or by differential protein expression for different thresholds on the effect size (see Figure S1). Overall, conclusions remain the same as for the original ground truth by p-values (compare Fig. 3 in the main text), with DrDimont performing better than the other two methods, and being predictive especially for top-ranked drugs.

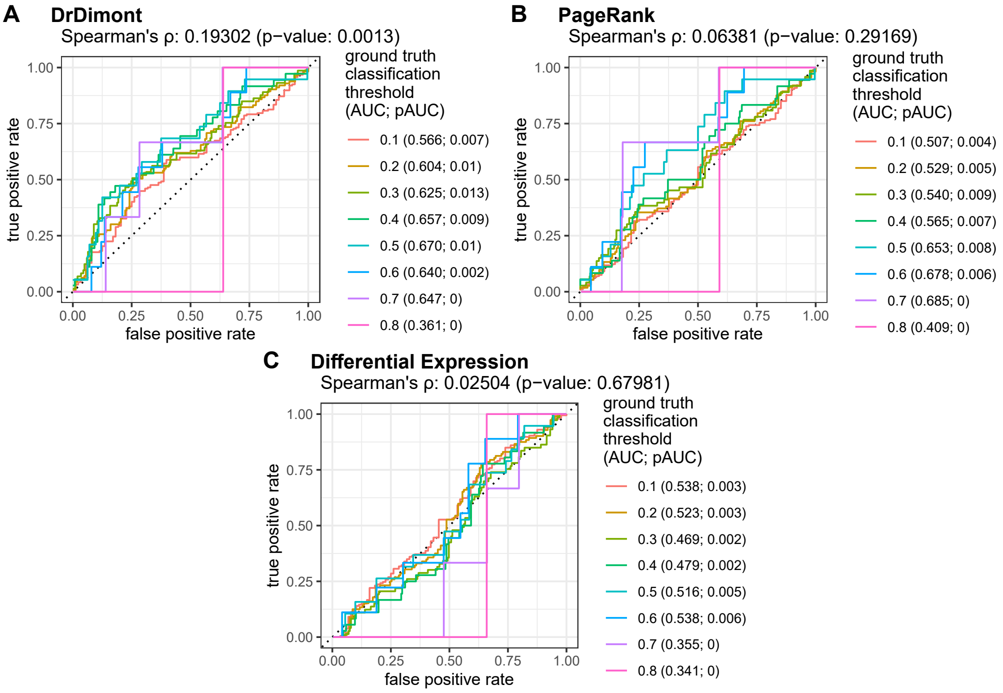

Figure S1: ROC curves for DrDimont's and alternative methods' prediction performance on the breast cancer dataset. Average effect size was used for ground truth drug ranking. (A) DrDimont's differential drug response (275 drugs). (B) Weighted PageRank of drug targets for differential drug response prediction (275 drugs). (C) Differential expression of drug target proteins for differential drug response prediction (274 drugs). In the legends, the values in brackets denote AUCs and pAUCs at given ground truth thresholds. The respective Spearman's ρ and the p-value are given at the top of each figure. Black dotted lines indicate theoretical ROC curves for random predictions.

2.2 Alternative reduction methods

DrDimont performs a reduction of the single-layer networks (see Methods, *Single-layer network generation*). By default, networks are reduced to maximize their scale-freeness, setting a goodness-of-fit threshold, R^2 (together with considerations on allowed network sizes). Other options implemented in DrDimont's framework are reductions by an approximate network density or average edge count. This means that the weakest network edges are removed until a specific density,

$$d = \frac{|E|}{|V| \cdot (|V|-1)},$$

that is the ratio of existing edges to possible edges in the network, or a specific mean number of edges per node

$$e = \frac{|E|}{|V|}$$

are reached. Here, E is the set of edges, V the set of vertices, and $|\cdot|$ denotes the cardinality of these sets. We investigated the effect of these alternative reduction schemes on DrDimont's performance for the TCGA + CPTAC dataset (see Figure S2). We find that for certain network densities d and average numbers of edges per node e, prediction results are comparable to the R^2-based reduction on scale-freeness. However, we observe that the prediction performance in terms of AUC and Spearman's correlation to the ground truth decreases for sparse ($e = 5$, $d = 0.001$) networks, but also for very densely connected networks ($e = 20$, $d = 0.007$).

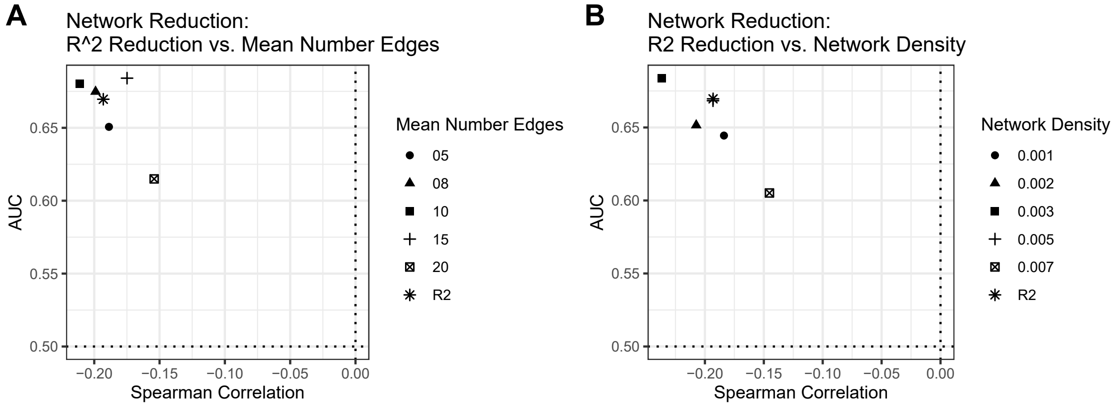

Figure S2: Effect of single-layer network reduction settings on DrDimont's performance. AUCs vs. Spearman's correlation for reduction upon (A) different average number of edges (5, 8, 10, 15, 20), (B) different network densities (0.001 - 0.007), compared to the prediction for reduction based on scale-freeness (R2). A ground truth threshold of 0.01 was used for AUC computation.

2.3 Integrated interaction score settings and differential drug prediction method

By default, we propagate local edge information in the integrated interaction scores by considering alternative paths up to a length of $L = 3$. Larger path lengths are computationally difficult due to the extremely high number of possible paths. For smaller path lengths, we observe reduced performance, especially in terms of the correlation between prediction and ground truth (symbols in Figure S3, TCGA+CPTAC breast cancer dataset). This shows that the local merging scheme we devised is beneficial.

Furthermore, we examined the effect of summarizing the edge information around drug targets for drug prediction (see *Differential drug response* in the Methods of the main text). By default, we employ the arithmetic mean of edge weights in the differential network over all edges that are incident to the drug's targets as differential drug response score of the drug ('mean (difference)'). This results in the fact that strong interactions that are present only in one of the contrasted groups can be balanced by alternative strong interactions to other nodes in the other group. Alternatively, we here consider other measures: (i) the median (instead of mean) over those edge weights ('median (difference)'), or (ii) absolute edge weights with mean ('mean (absolute difference)'), or (iii) median ('median (absolute difference)') (colors in Figure S3). We find that our default measure performs decisively better than the other three, both in terms of AUC and correlation. Thereby, both measures that do not consider edge weights in absolute terms are better, and taking the mean is (at least slightly) better than taking the median.

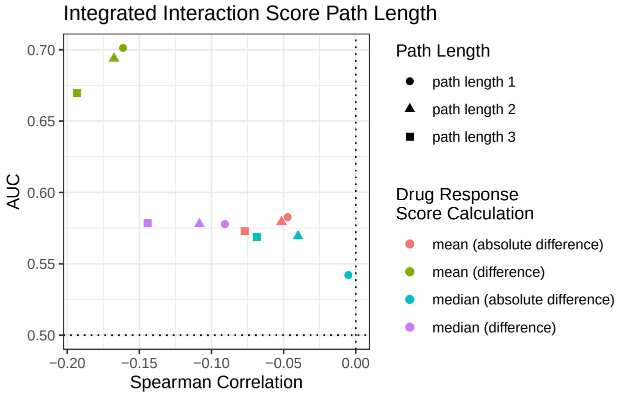

Figure S3: Integrated interaction score settings and differential drug computation. We examined DrDimont's performance, AUC vs. correlation between prediction and ground truth (CTRP), on the TCGA + CPTAC dataset for different settings. We consider (i) the path length for computing the integrated interaction score: 3 (default, square), 2 (triangle), 1 (no propagation of local information, circle), and (ii) the summarizing scheme for edge weights incident to a drug's targets in the differential network: mean of absolute differential edge weights (red), mean of differential edge weights (default, green), median of absolute differential edge weights (blue), and median of differential edge weights (purple).

2.4 ROC curves for data layer ablation study

We here provide ROC curves for the data layer ablation study on the breast cancer patient dataset (from TCGA and CPTAC) with performance indicators from Table 1 from the main text. Default setting was to include the mRNA, protein, phosphosite layers (red in all plots in Figure S4). Apart from different combinations of these three layers, we assessed the impact of including data from one of two metabolomics studies [4, 22]. Please refer to the main text for the description of the results.

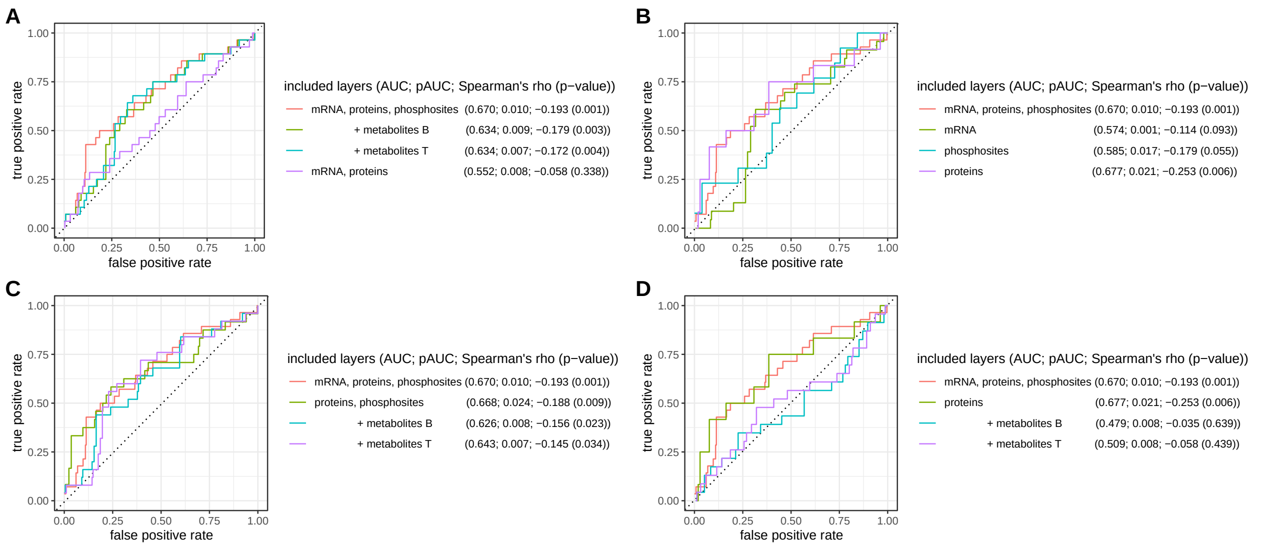

Figure S4: ROC curves for the performance of DrDimont when supplied different combinations of data layers for the TCGA + CPTAC breast cancer dataset. The default setting with mRNA, protein, phosphosite layers is given in each plot (red). (A) Addition of metabolomics, or removal of phosphosite data. (B) Single-layer prediction. (C) Protein and phosphosite layer, combined with metabolomics. (D) Protein layer, combined with metabolomics. Comparison against ground truth from CTRP; AUC and pAUC for a ground truth threshold of 0.01. B: Budczies dataset [4], T: Terunuma dataset [22]. Please refer to Table 1 (main text) for numbers of drugs with predictions in each setting.

2.5 Drugs lacking predictions for data layers

We examined in more detail which drugs can be predicted as differential by DrDimont depending on the data layers that were provided. We focus on the case of using the protein layer only (best performing) or using the protein plus mRNA layers (strong decrease in performance). In particular, when using only the protein layer, we find that 159 of 275 drugs from CTRP that had drug targets in our molecular networks could not be predicted as differential or not by DrDimont. This happens if there are no edges incident to drug targets in either of the two compared conditions. Some of the most differential drugs (as indicated by CTRP ground truth) were among those (see Figure S5A). In contrast, when adding the mRNA layer, all 275 proteins receive DrDimont scores, but all but three of the ones without predictions for the protein layer only are zero (see Figure S5C). For drugs with DrDimont predictions in both settings, results are very similar (see Figure S5D).

The four drugs with highest differential predictions in both settings were AZ-3146, tamatinib, SB-743921, ibrutinib. The three drugs with high differential ground truth were AZ-D7762, MK1775, GSK461364. The drug with highest differential response in ground truth that received differential predictions in both settings was momelotinib (DrDimont's drug response scores: 0.66 (protein), 0.63 (protein + mRNA)).

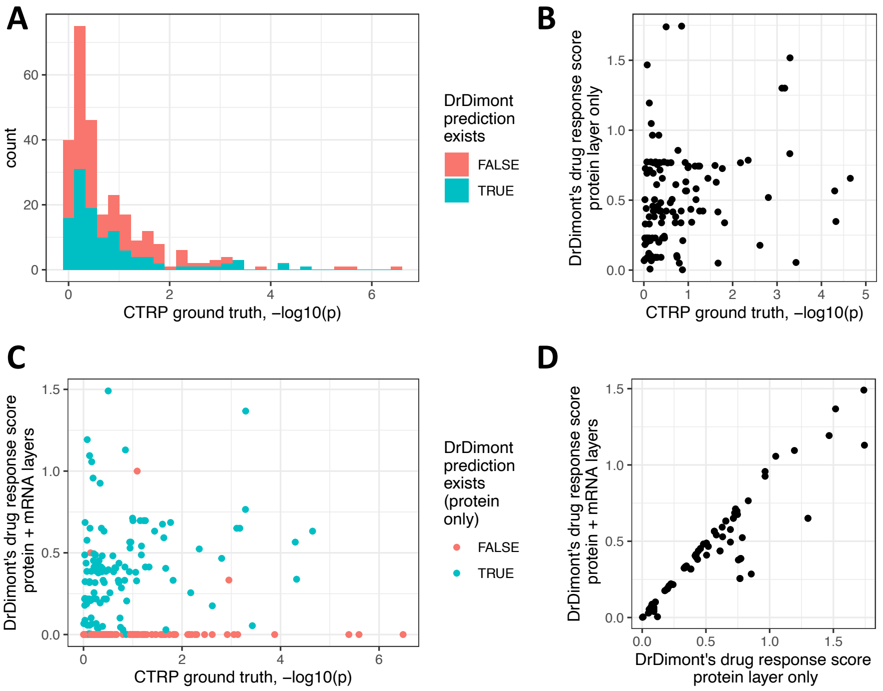

Figure S5: Effects of using only the protein layer or protein and mRNA layers on DrDimont's predictions. (A) Histogram of ground truth predictions for CTRP (negative log10 of the Mann-Whitney-U p-values) for 275 drugs. The color distinguishes whether DrDimont could derive predictions for the drug from the protein layer only (116 drugs, blue) or not (159 drug, red). (B) DrDimont's differential drug response score using only the protein layer vs. CTRP ground truth differential drug score predictions for 116 drugs. (C) DrDimont's differential drug response score using the protein and mRNA layers vs. CTRP ground truth differential drug score predictions for all 275 drugs. Color indicates whether DrDimont could derive predictions from the protein layer only (as in A). (D) DrDimont's differential drug response scores using the protein layer and mRNA layers vs. using only the protein layer for 116 drugs.

2.6 Alternative ground truth measurements from GDSC

In our analyses, we focused on CTRP [20] as differential drug response ground truth because it delivered values for many drugs (490 measured, 275 with predictions in our framework). In order to assess the impact of this selected ground truth, we also employed the processed GDSC drug sensitivity dataset [29] that delivers data for 42 breast cancer cell lines (29 ER-, 13 ER+) and 198 drugs. From that, we used the area under the drug response curves (AUC) as drug sensitivity information. We retrieved the drug target information from the compound annotation file and matched protein names or target descriptions to Gene Symbols using the GeneCards resource. We only employed data for a drug if it was measured at least three times for each ER+ and ER- cell lines. We determined the differential drug response between ER+ and ER- for each drug by a Mann-Whitney-U test comparing sensitivity in ER+ cell lines vs. sensitivity in ER- cell lines. Thus, we obtained ground truth for 190 drugs from GDSC. For the dataset with TCGA mRNA, CPTAC proteomics and phosphosites, DrDimont predicted differential response for 101 drugs. A comparison between the ground truth from GDSC and CTRP and performance of DrDimont for them is shown in Figure S6. It becomes clear that the ground truths do not overlap very well.

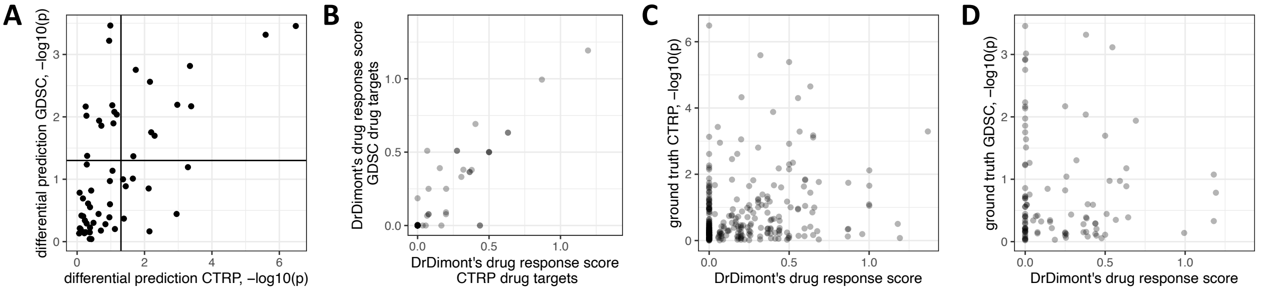

Figure S6: CTRP vs. GDSC ground truth for differential drug response. (A) Differential response ground truth negative logarithmized p-values for 57 drugs that overlap in the datasets from CTRP and GDSC. Solid lines indicate p-values of 0.05. (B) DrDimont's drug response scores for the TCGA + CPTAC breast cancer dataset (mRNA, protein, phosphosites) for 53 drugs overlapping between CTRP and GDSC. Different scores stem from different drug target definitions, 4 of the 57 drugs from (A) did not have drug targets in our network. (C) DrDimont's drug response scores compared to CTRP ground truth for 275 drugs. Dataset as in B. Performance at a 0.01 ground-truth threshold: AUC 0.67, pAUC 0.01; Spearman's ρ -0.193, p-value 0.001. (D) DrDimont's drug response scores compared to GDSC ground truth for 101 drugs. Dataset as in B. Performance at a 0.01 ground-truth threshold: AUC 0.4, pAUC 0; Spearman's ρ 0.01, p-value 0.9.

2.7 Explanations for DrDimont's top-predicted drugs and single drug targets

DrDimont allows for retrieving molecular explanations for the derived drug response score. In addition to explanations for dinaciclib's drug response (Figure 4), we here show the analyses for the top-differential predicted drugs for the reduced TCGA dataset with mRNA, protein, phosphosite layers (see Figure 3B), SB-743921, tamatinib, ibrutinib (Figure S7A-C). There is only one drug target for each drug present in the DrDimont-derived differential network. For all three drugs, the large differential drug response scores stem from protein-protein edges with large positive weights around the drug targets that are present in one condition (ER+ or ER-) but not in the other condition (integrated interaction scores of zero).

In addition, we performed a systematic analysis of all 189 protein nodes that are drug targets in our CTRP dataset (Figure S7D). For each protein, we computed the mean differential integrated interaction score of the edges adjacent to its node in the differential network. This measure is assessing how different the drug targets are wired comparing the two conditions. It is tightly related to DrDimont's differential drug response score: For drugs with only one target in the network, it is the (signed) differential drug response score; for drugs with more than one target, they enter in a weighted form according to the number of adjacent edges of each drug target. In fact, the three top-scoring drugs (see Figure S7A-C) target three of the highest-scoring proteins in this analysis. If a drug was to target multiple proteins that score highly with the same sign in this analysis (e.g., BCR and UGCG along with KIF11), its differential drug response score would be even larger.

This analysis highlights the potential of using DrDimont's framework for gene-based differential analysis, e.g., for the detection of new biomarkers.

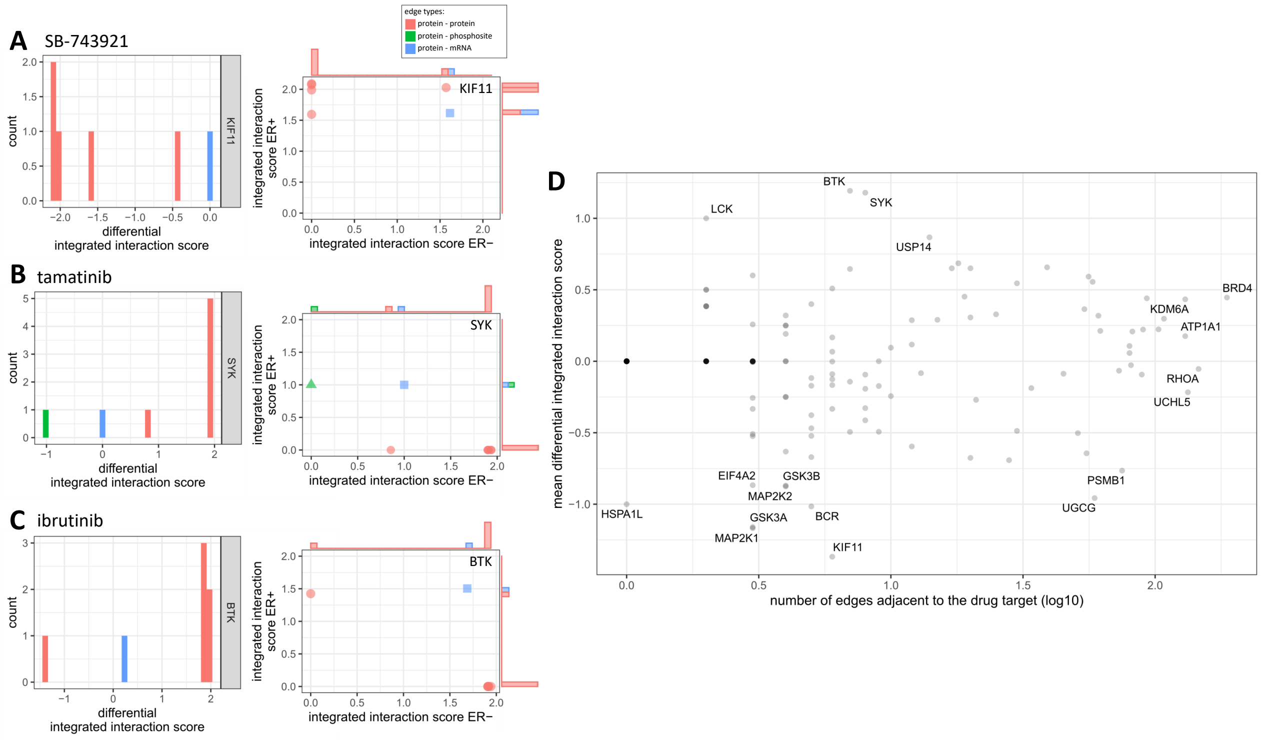

Figure S7: Molecular explanations for DrDimont's predictions. A-C: Explanations for the drugs SB-743921, tamatinib, ibrutinib with the largest differential drug response scores in our analyses of the TCGA dataset. The histograms (left panels) show the differential integrated interaction scores for all edges adjacent to the drug target of each drug; the scatter plots (right panels) resolve the integrated interaction scores for the two different conditions. D: Mean integrated interactions scores for edges adjacent to 189 protein nodes which are drug targets in CTRP and appear in the differential network. In addition, the x-axis yields the number of adjacent edges for each protein. Proteins with largest absolute mean differential integrated interaction scores (> 0.75) are annotated, as well as those with the largest numbers of adjacent edges (> 125). These results rely on the reduced TCGA dataset containing proteomics, phosphosites and mRNA layers.

2.8 Performance for unreduced and other datasets

In addition to the TCGA + CPTAC dataset, we applied DrDimont to two other datasets for ER+ vs. ER- breast cancer: (i) a second independent breast cancer patient study with mRNA, protein, phosphosites [11] (‘Krug’), and (ii) cell line data from the Cancer Cell Line Encyclopedia (CCLE) with mRNA, proteins and metabolomics [13, 16]. The mRNA, protein and phosphosite data for the Krug dataset was derived from the Supplementary Table 2 of Krug et al. [11]. The breast cancer cell line data was downloaded from the CCLE [10, 16, 13].

For the Krug dataset, we only employed samples with tumor purity (according to the metadata) > 0.5; the ER status of the samples was reported in the metadata file [11]. For the cell line data, we used estrogen receptor status annotation from the CCLE resource.

Both mRNA datasets stem from RNAseq. We neglected mRNAs with more than 90% of zero measurements over the samples within a condition. Proteins and phosphosites with more than 20% of missing values over the samples of a condition were removed. DrDimont’s performance was compared to predictions derived from differential protein expression analysis (see Methods, *Differential protein expression*).

In addition, we re-analyzed the TCGA + CPTAC (‘TCGA’) dataset without genetic feature reduction, and no genetic feature selection was performed for the other two datasets either. See Table S1 for DrDimont’s settings, sample sizes for each subgroup of the datasets, and generated network sizes.

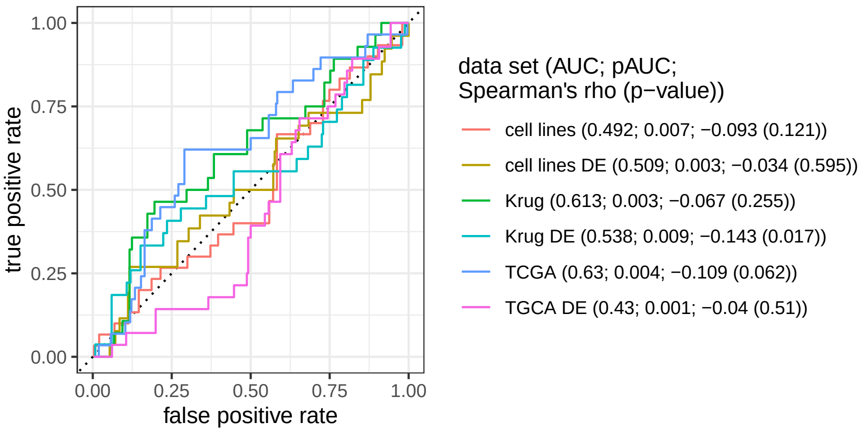

Figure S8: DrDimont’s differential drug prediction results for three different datasets, compared to prediction by differential expression (‘DE’): breast cancer cell lines (‘cell lines’), an alternative breast cancer patient study (‘Krug’), and TCGA + CPTAC breast cancer patients (’TCGA’). Genetic features were not reduced. ROC analysis was performed with a ground-truth threshold of 0.01 for the CTRP ground truth. The AUC, pAUC and Spearman’s ρ and its significance (p-value) are described in the legend. Predictions are derived for 278 drugs in the cell lines dataset, 295 drugs in the Krug dataset, and 292 drugs in the entire TCGA dataset.

For the cell line data, DrDimont’s prediction showed a performance close to random in terms of AUC (0.492), but elevated pAUC (0.007 vs. 0.005 expected for random predictions) and also a small correlation to the ground truth prediction (Spearman’s ρ -0.093, p-value 0.12). The drug response prediction by differential expression performed worse with even lower correlation (-0.034) and decisively lower pAUC (0.003). Reasons for the bad performance on the cell line dataset could be the decisively lower sample size that characterizes each subgroup. In addition, we lack the phosphosite layer for this dataset that had shown to confer robustness to the results in our TCGA + CPTAC dataset.

For the Krug dataset, DrDimont’s predictions yielded an AUC of 0.61 and an insignificant Spearman’s ρ of -0.07 (p-value 0.255). Top-ranked results were not retrieved particularly frequently (pAUC 0.003). For differential drug response by differential protein expression in the Krug dataset, lower AUC (0.538), but significant correlation (-0.14, p-value 0.017) and a higher pAUC than expected by random predictions (0.009) were obtained. Therefore, in this dataset, it seems that differential protein expression is a better marker for differential drug response than network-based interaction analysis. This suggests that including differential expression into the analysis approaches might be beneficial under certain circumstances.

DrDimont’s drug response scores of the unreduced TCGA dataset resulted in slightly worse predictions than for the reduced dataset (AUC 0.63 for ground-truth threshold 0.01, Spearman’s ρ -0.11, p-value 0.062), and especially lacked enrichment of positive predictions for top-ranked results (pAUC 0.004). Thus, feature selection seems beneficial for differential drug response.

References

[1] J. Allaire, Y. Xie, J. McPherson, J. Luraschi, K. Ushey, A. Atkins, H. Wickham, J. Cheng, W. Chang, and R. Iannone. rmarkdown: Dynamic documents for R. `https://github.com/rstudio/rmarkdown`, 2022. R package version 2.13.

[2] D. Attali and C. Baker. ggextra: Add marginal histograms to 'ggplot2', and more 'ggplot2' enhancements. `https://CRAN.R-project.org/package=ggExtra`, 2022. R package version 0.10.0.

[3] S. M. Bache and H. Wickham. magrittr: A forward-pipe operator for R. `https://CRAN.R-project.org/package=magrittr`, 2022. R package version 2.0.3.

[4] J. Budczies, S. F. Brockmöller, B. M. Muller, D. K. Barupal, C. Richter-Ehrenstein, A. Kleine-Tebbe, J. L. Griffin, M. Oresic, M. Dietel, C. Denkert, and O. Fiehn. Comparative metabolomics of estrogen receptor positive and estrogen receptor negative breast cancer: alterations in glutamine and beta-alanine metabolism. *J Proteomics*, 94:279–88, 2013.

[5] G. Csardi and T. Nepusz. The igraph software package for complex network research. `https://igraph.org`, 2006.

[6] C. da Costa-Luis, S. K. Larroque, K. Altendorf, H. Mary, richardsheridan, M. Korobov, N. Yorav-Raphael, I. Ivanov, M. Bargull, N. Rodrigues, G. CHEN, A. Lee, C. Newey, James, J. Coales, M. Zugnoni, M. D. Pagel, mjstevens777, M. Dektyarev, A. Rothberg, Alexander, D. Panteleit, F. Dill, FichteFoll, G. Sturm, HeoHeo, H. van Kemenade, J. McCracken, MapleCCC, and M. Nordlund. tqdm: A fast, extensible progress bar for Python and CLI. `https://doi.org/10.5281/zenodo.5202772`, Aug. 2021.

[7] F. Harrell Jr. Hmisc: Harrell miscellaneous. `https://CRAN.R-project.org/package=Hmisc`, 2021.

[8] C. R. Harris, K. J. Millman, S. J. van der Walt, R. Gommers, P. Virtanen, D. Cournapeau, E. Wieser, J. Taylor, S. Berg, N. J. Smith, R. Kern, M. Picus, S. Hoyer, M. H. van Kerkwijk, M. Brett, A. Haldane, J. F. del Río, M. Wiebe, P. Peterson, P. Gérard-Marchant, K. Sheppard, T. Reddy, W. Weckesser, H. Abbasi, C. Gohlke, and T. E. Oliphant. Array programming with NumPy. *Nature*, 585(7825):357–362, Sept. 2020.

[9] L. Henry and H. Wickham. rlang: Functions for base types and core R and 'tidyverse' features. `https://CRAN.R-project.org/package=rlang`, 2022. R package version 1.0.2.

[10] F. Iorio, T. A. Knijnenburg, D. J. Vis, G. R. Bignell, M. P. Menden, M. Schubert, N. Aben, E. Goncalves, S. Barthorpe, H. Lightfoot, T. Cokelaer, P. Greninger, E. van Dyk, H. Chang, H. de Silva, H. Heyn, X. Deng, R. K. Egan, Q. Liu, T. Mironenko, X. Mitropoulos, L. Richardson, J. Wang, T. Zhang, S. Moran, S. Sayols, M. Soleimani, D. Tamborero, N. Lopez-Bigas, P. Ross-Macdonald, M. Esteller, N. S. Gray, D. A. Haber, M. R. Stratton, C. H. Benes, L. F. A. Wessels, J. Saez-Rodriguez, U. McDermott, and M. J. Garnett. A landscape of pharmacogenomic interactions in cancer. *Cell*, 166(3):740–754, 2016.

[11] K. Krug, E. J. Jaehnig, S. Satpathy, L. Blumenberg, A. Karpova, M. Anurag, G. Miles, P. Mertins, Y. Geffen, L. C. Tang, D. I. Heiman, S. Cao, Y. E. Maruvka, J. T. Lei, C. Huang, R. B. Kothadia, A. Colaprico, C. Birger, J. Wang, Y. Dou, B. Wen, Z. Shi, Y. Liao, M. Wiznerowicz, M. A. Wyczalkowski, X. S. Chen, J. J. Kennedy, A. G. Paulovich, M. Thiagarajan, C. R. Kinsinger, T. Hiltke, E. S. Boja, M. Mesri, A. I. Robles, H. Rodriguez, T. F. Westbrook, L. Ding, G. Getz, K. R. Clauser, D. Fenyö, K. V. Ruggles, B. Zhang, D. R. Mani, S. A. Carr, M. J. Ellis, and M. A. Gillette. Proteogenomic landscape of breast cancer tumorigenesis and targeted therapy. *Cell*, 183(5):1436–1456.e31, 2020.

[12] P. Langfelder and S. Horvath. WGCNA: an R package for weighted correlation network analysis. *BMC Bioinformatics*, 9:559, 2008.

[13] H. Li, S. Ning, M. Ghandi, G. V. Kryukov, S. Gopal, A. Deik, A. Souza, K. Pierce, P. Keskula, D. Hernandez, J. Ann, D. Shkoza, V. Apfel, Y. Zou, F. Vazquez, J. Barretina, R. A. Pagliarini, G. G. Galli, D. E. Root, W. C. Hahn, A. Tsherniak, M. Giannakis, S. L. Schreiber, C. B. Clish, L. A. Garraway, and W. R. Sellers. The landscape of cancer cell line metabolism. *Nat Med*, 25(5):850–860, 2019.

[14] P. Moritz, R. Nishihara, S. Wang, A. Tumanov, R. Liaw, E. Liang, M. Elibol, Z. Yang, W. Paul, M. I. Jordan, and I. Stoica. Ray: A distributed framework for emerging AI applications. In *Proceedings of the 13th USENIX Conference on Operating Systems Design and Implementation*, OSDI'18, page 561–577, USA, 2018. USENIX Association.

[15] K. Müller and H. Wickham. tibble: Simple data frames. `https://CRAN.R-project.org/package=tibble`, 2021. R package version 3.1.6.

[16] D. P. Nusinow, J. Szpyt, M. Ghandi, C. M. Rose, E. R. McDonald, M. Kalocsay, J. Jané-Valbuena, E. Gelfand, D. K. Schweppe, M. Jedrychowski, J. Golji, D. A. Porter, T. Rejtar, Y. K. Wang, G. V. Kryukov, F. Stegmeier, B. K. Erickson, L. A. Garraway, W. R. Sellers, and S. P. Gygi. Quantitative proteomics of the Cancer Cell Line Encyclopedia. *Cell*, 180(2):387–402.e16, 2020.

[17] M. Papadakis, M. Tsagris, M. Dimitriadis, S. Fafalios, I. Tsamardinos, M. Fasiolo, G. Borboudakis, J. Burkardt, C. Zou, K. Lakiotaki, and C. Chatzipantsiou. Rfast: A collection of efficient and extremely fast R functions. `https://CRAN.R-project.org/package=Rfast`, 2022. R package version 2.0.6.

[18] Python Core Team. Python: A dynamic, open source programming language. `https://www.python.org/`, 2021. Python version 3.9.6.

[19] R Core Team. R: A language and environment for statistical computing. `https://www.R-project.org/`, 2021.

[20] M. G. Rees, B. Seashore-Ludlow, J. H. Cheah, D. J. Adams, E. V. Price, S. Gill, S. Javaid, M. E. Coletti, V. L. Jones, N. E. Bodycombe, C. K. Soule, B. Alexander, A. Li, P. Montgomery, J. D. Kotz, C. S.-Y. Hon, B. Munoz, T. Liefeld, V. Dančík, D. A. Haber, C. B. Clish, J. A. Bittker, M. Palmer, B. K. Wagner, P. A. Clemons, A. F. Shamji, and S. L. Schreiber. Correlating chemical sensitivity and basal gene expression reveals mechanism of action. *Nature Chemical Biology*, 12:109, 2015.

[21] D. Szklarczyk, A. Santos, C. von Mering, L. J. Jensen, P. Bork, and M. Kuhn. STITCH 5: augmenting protein-chemical interaction networks with tissue and affinity data. *Nucleic Acids Res*, 44(D1):D380–4, 2016.

[22] A. Terunuma, N. Putluri, P. Mishra, E. A. Mathé, T. H. Dorsey, M. Yi, T. A. Wallace, H. J. Issaq, M. Zhou, J. K. Killian, H. S. Stevenson, E. D. Karoly, K. Chan, S. Samanta, D. Prieto, T. Y. Hsu, S. J. Kurley, V. Putluri, R. Sonavane, D. C. Edelman, J. Wulff, A. M. Starks, Y. Yang, R. A. Kittles, H. G. Yfantis, D. H. Lee, O. B. Ioffe, R. Schiff, R. M. Stephens, P. S. Meltzer, T. D. Veenstra, T. F. Westbrook, A. Sreekumar, and S. Ambs. MYC-driven accumulation of 2-hydroxyglutarate is associated with breast cancer prognosis. *J Clin Invest*, 124(1):398–412, 2014.

[23] H. Wickham. *ggplot2: Elegant Graphics for Data Analysis*. Springer, 2016.

[24] H. Wickham. stringr: Simple, consistent wrappers for common string operations. `https://CRAN.R-project.org/package=stringr`, 2019. R package version 1.4.0.

[25] H. Wickham, R. Francois, L. Henry, and K. Müller. dplyr: A grammar of data manipulation. `https://CRAN.R-project.org/package=dplyr`, 2021. R package version 1.0.7.

[26] H. Wickham and M. Girlich. tidyr: Tidy messy data. `https://CRAN.R-project.org/package=tidyr`, 2022. R package version 1.2.0.

[27] H. Wickham, J. Hester, and J. Bryan. readr: Read rectangular text data. `https://CRAN.R-project.org/package=readr`, 2022. R package version 2.1.2.

[28] Y. Xie. knitr: A general-purpose package for dynamic report generation in R. `https://yihui.org/knitr/`, 2022. R package version 1.38.

[29] W. Yang, J. Soares, P. Greninger, E. J. Edelman, H. Lightfoot, S. Forbes, N. Bindal, D. Beare, J. A. Smith, I. R. Thompson, S. Ramaswamy, P. A. Futreal, D. A. Haber, M. R. Stratton, C. Benes, U. McDermott, and M. J. Garnett. Genomics of Drug Sensitivity in Cancer (GDSC): a resource for therapeutic biomarker discovery in cancer cells. *Nucleic Acids Research*, 41(D1):D955–D961, 2013.
